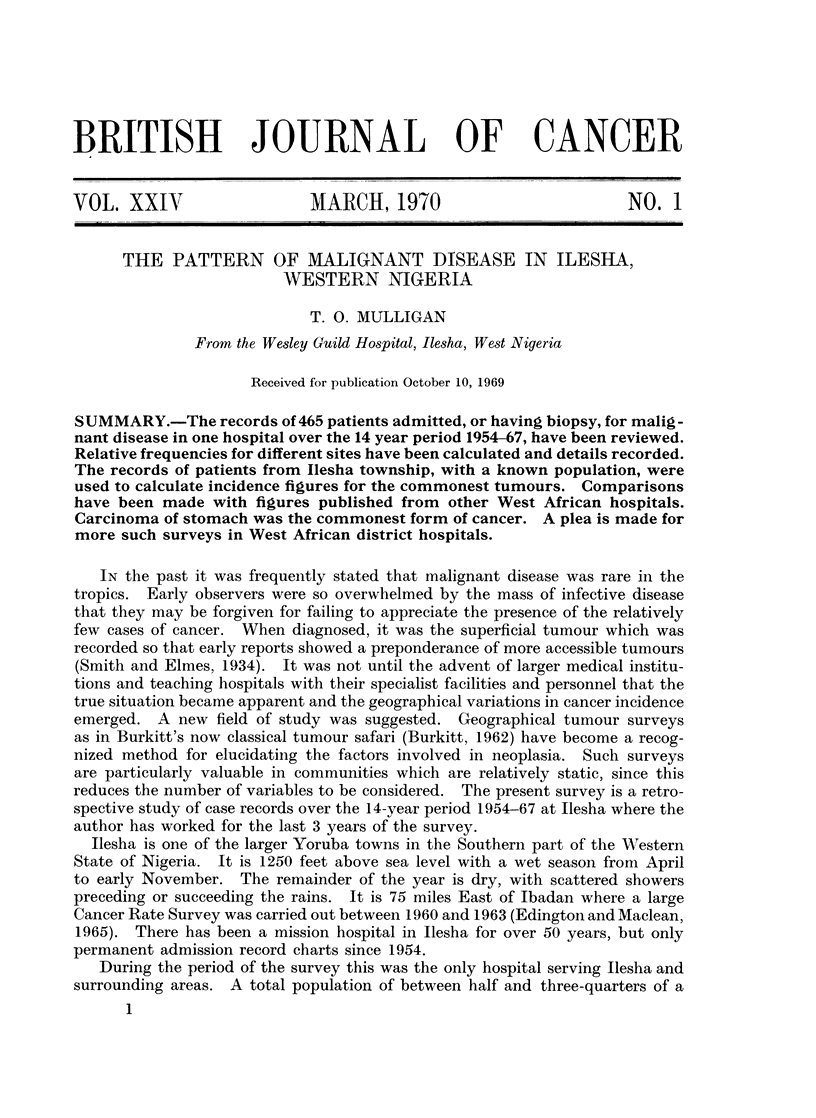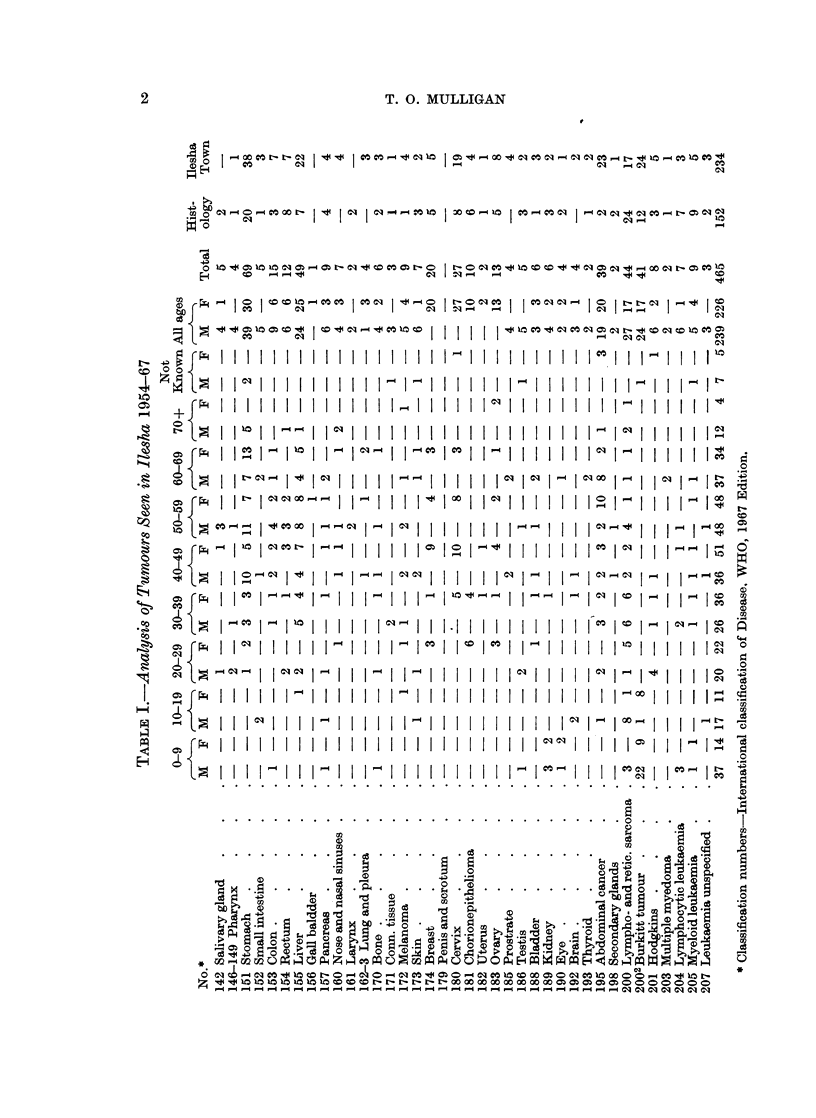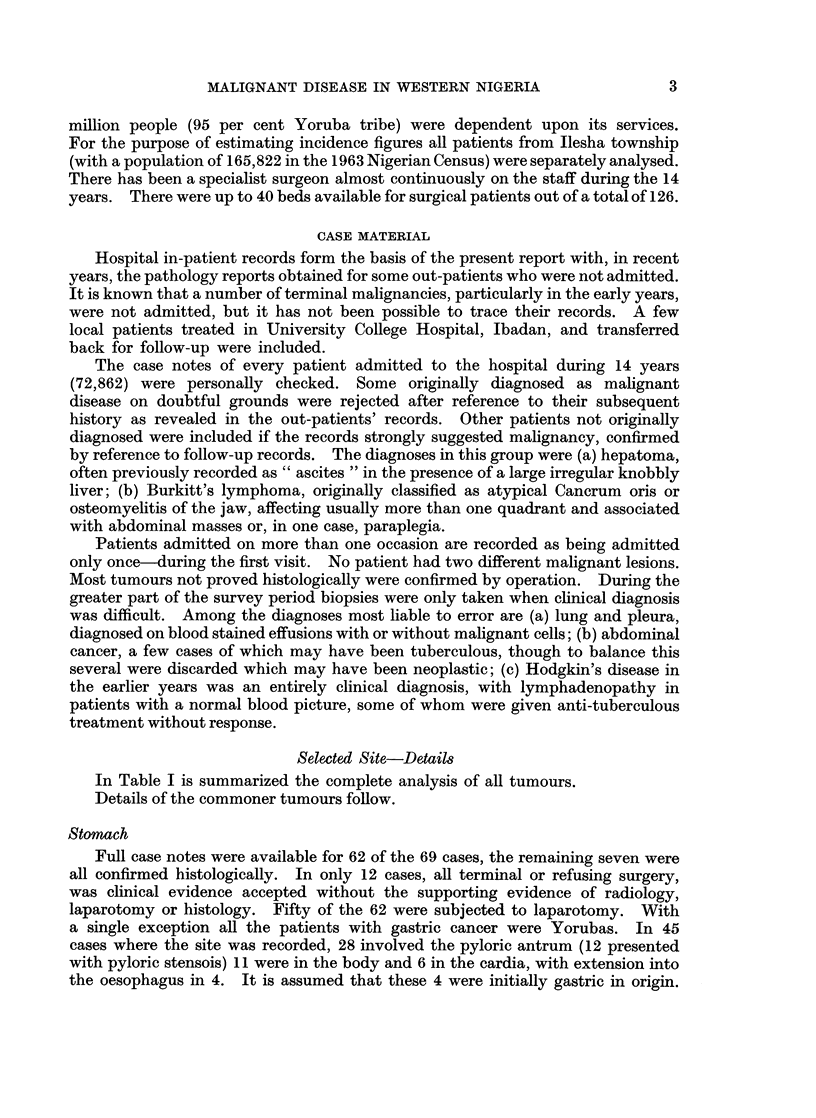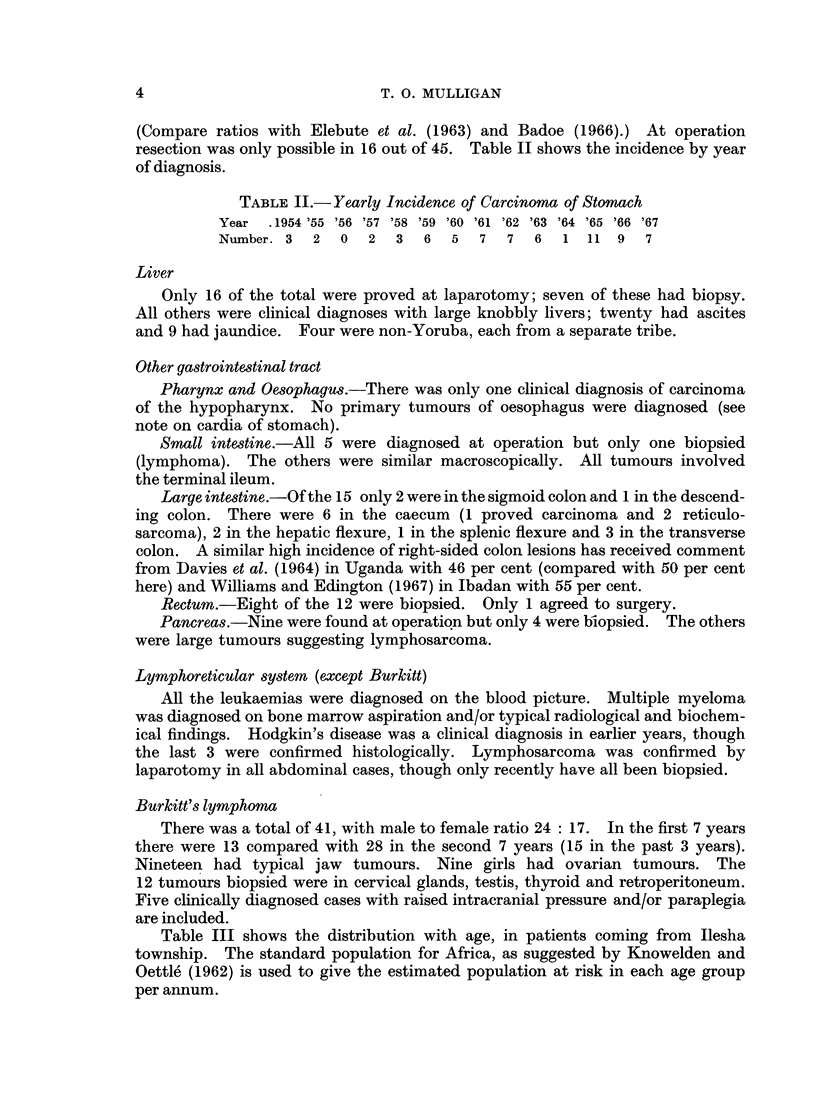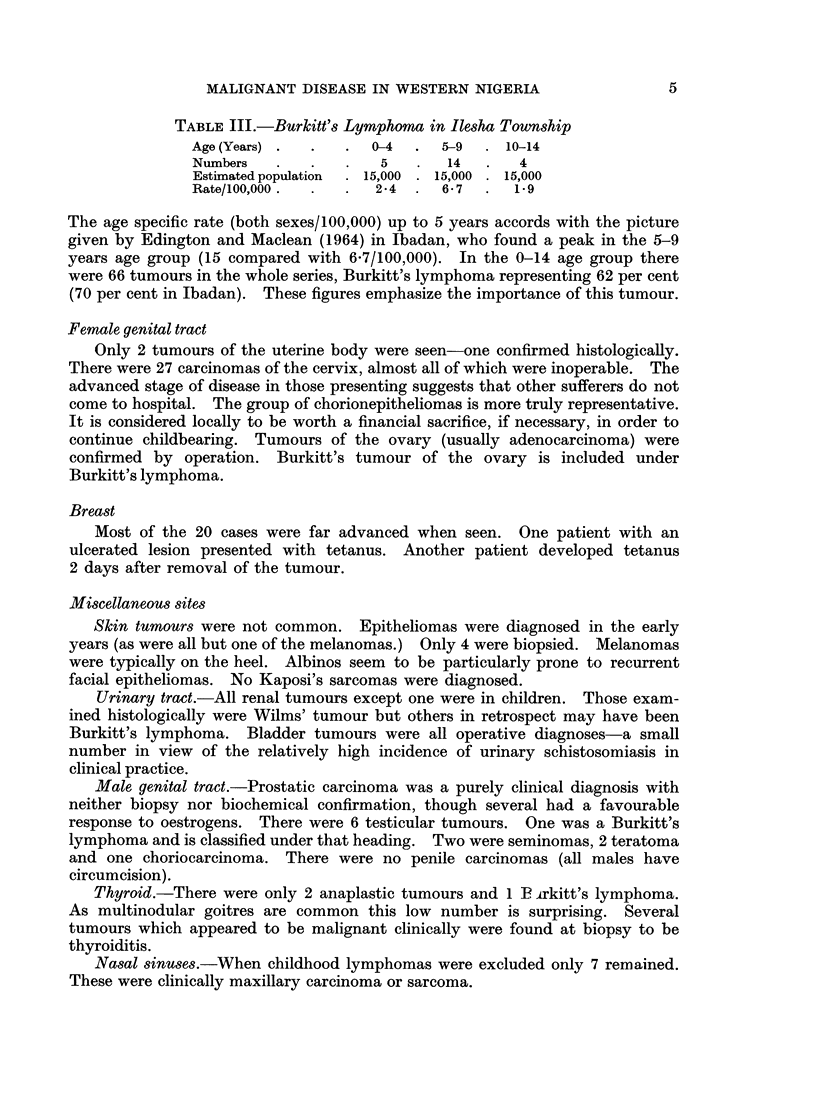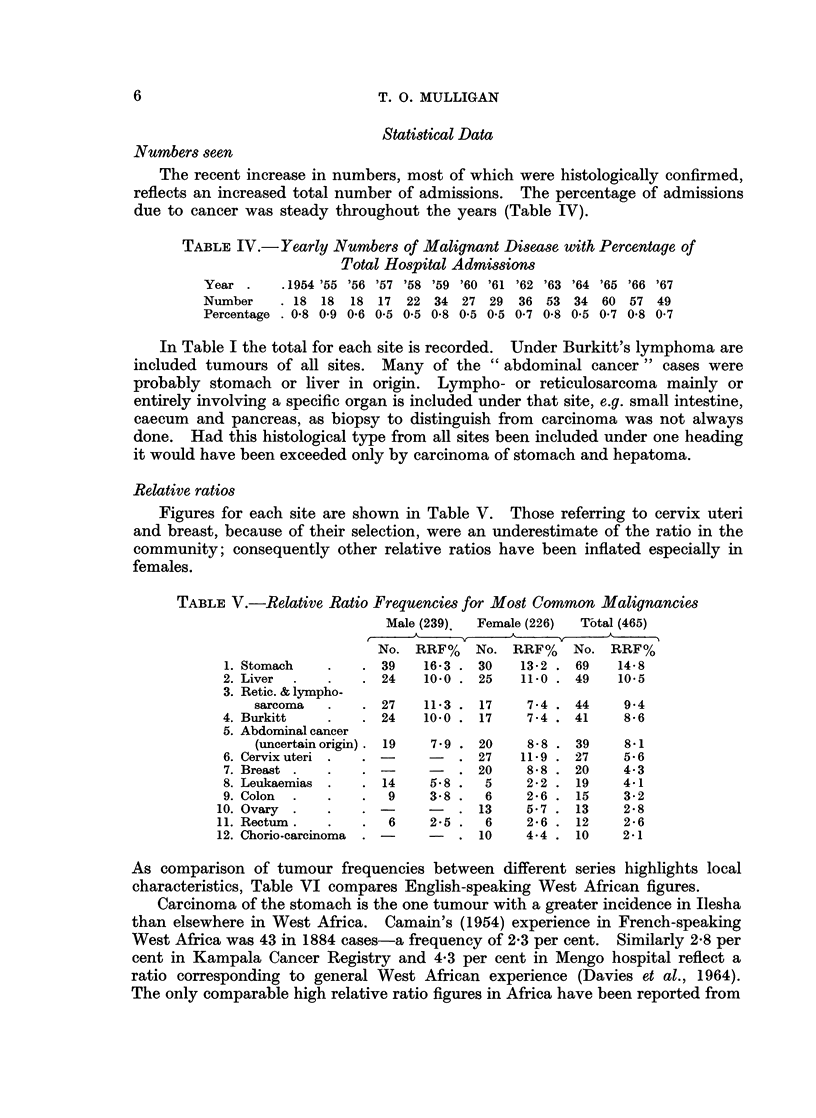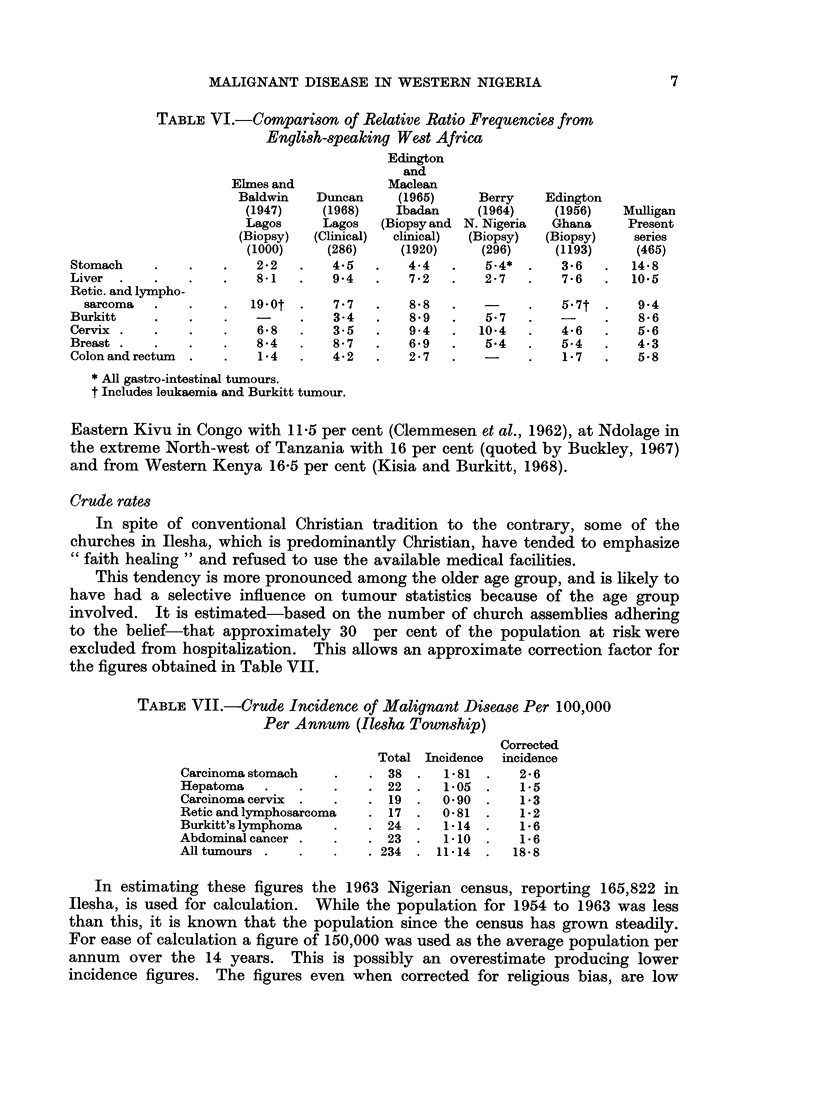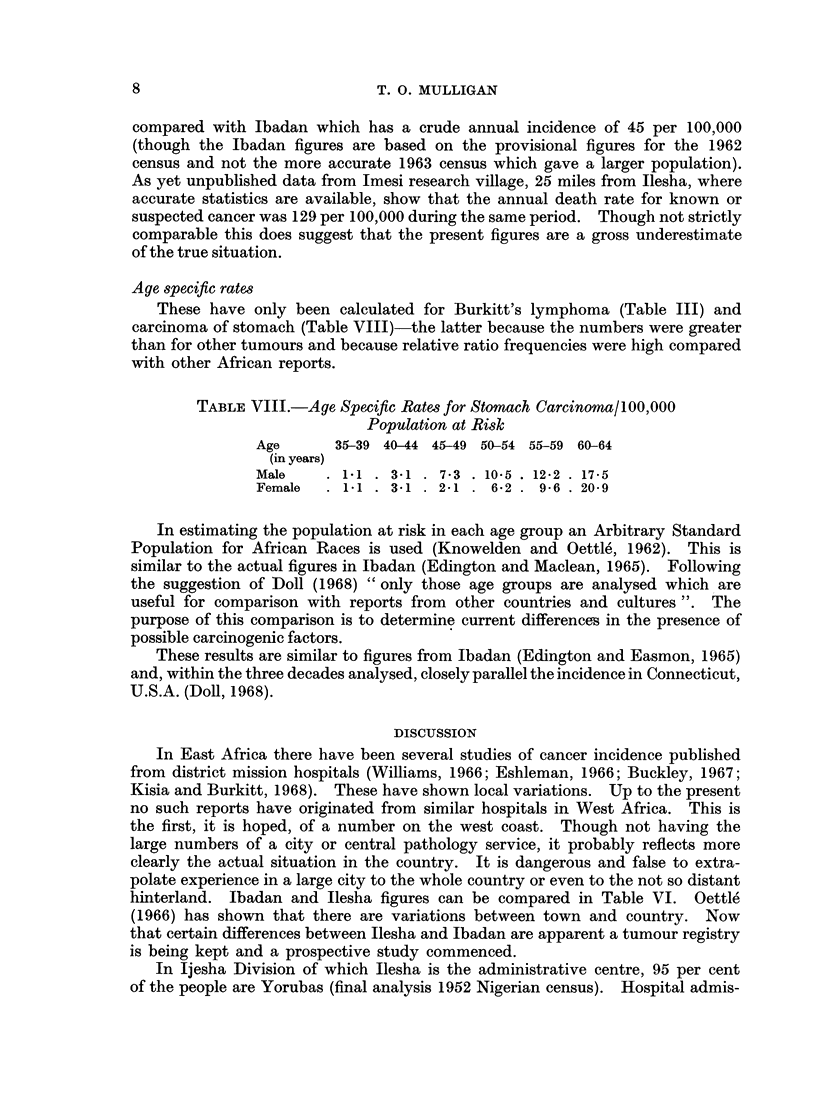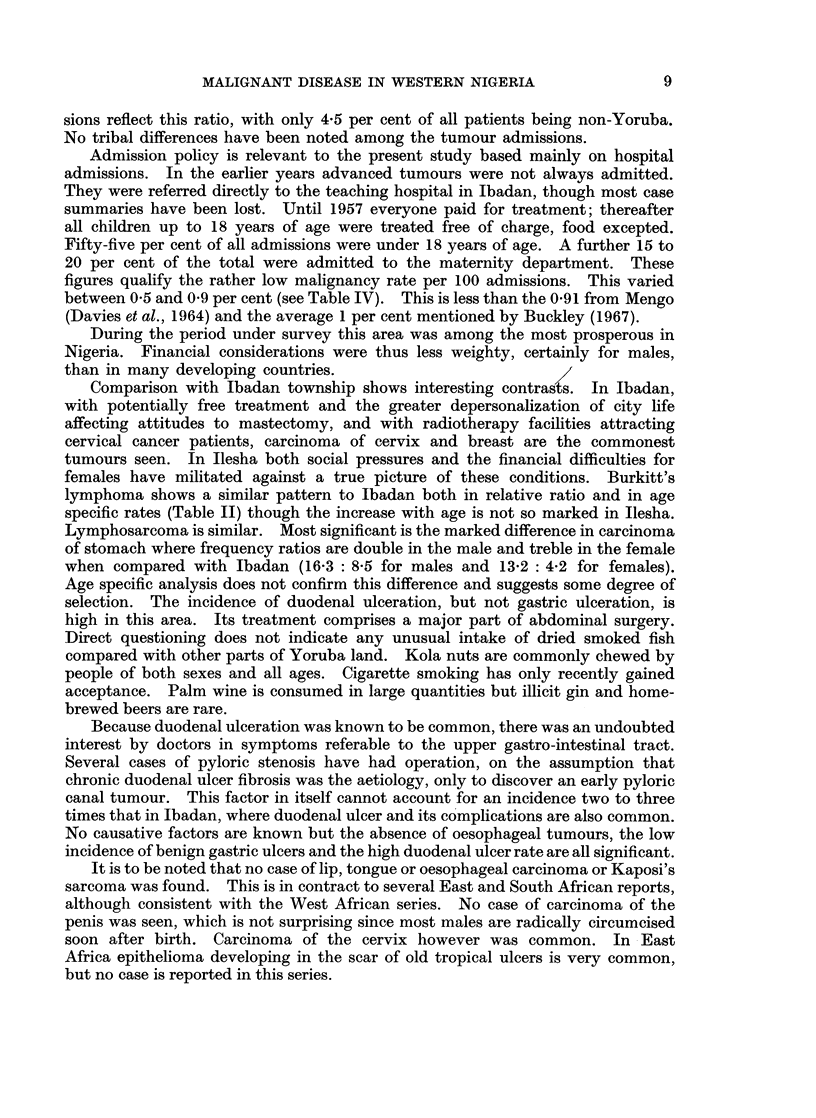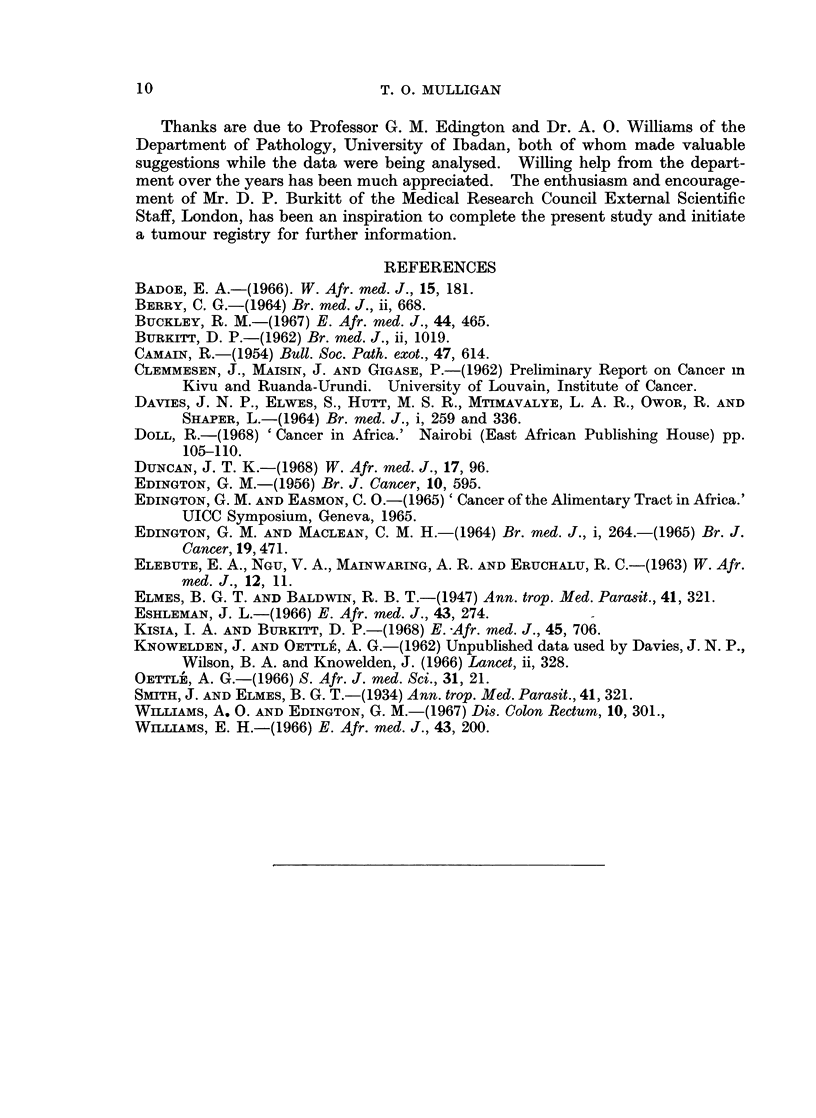# The Pattern of Malignant Disease in Ilesha, Western Nigeria

**DOI:** 10.1038/bjc.1970.1

**Published:** 1970-03

**Authors:** T. O. Mulligan

## Abstract

The records of 465 patients admitted, or having biopsy, for malignant disease in one hospital over the 14 year period 1954-67, have been reviewed. Relative frequencies for different sites have been calculated and details recorded. The records of patients from Ilesha township, with a known population, were used to calculate incidence figures for the commonest tumours. Comparisons have been made with figures published from other West African hospitals. Carcinoma of stomach was the commonest form of cancer. A plea is made for more such surveys in West African district hospitals.


BRITISH JOURNAL OF CANCER

VOL. XXIV MARCH, 1970 NO. 1

### THE PATTERN OF MALIGNANT DISEASE IN ILESHA, WESTERN NIGERIA

T. O. MULLIGAN

*From the Wesley Guild Hospital, Ilesha, West Nigeria*

Received for publication October 10, 1969

**SUMMARY.—The records of 465 patients admitted, or having biopsy, for malignant disease in one hospital over the 14 year period 1954–67, have been reviewed. Relative frequencies for different sites have been calculated and details recorded. The records of patients from Ilesha township, with a known population, were used to calculate incidence figures for the commonest tumours. Comparisons have been made with figures published from other West African hospitals. Carcinoma of stomach was the commonest form of cancer. A plea is made for more such surveys in West African district hospitals.**

IN the past it was frequently stated that malignant disease was rare in the tropics. Early observers were so overwhelmed by the mass of infective disease that they may be forgiven for failing to appreciate the presence of the relatively few cases of cancer. When diagnosed, it was the superficial tumour which was recorded so that early reports showed a preponderance of more accessible tumours (Smith and Elmes, 1934). It was not until the advent of larger medical institutions and teaching hospitals with their specialist facilities and personnel that the true situation became apparent and the geographical variations in cancer incidence emerged. A new field of study was suggested. Geographical tumour surveys as in Burkitt's now classical tumour safari (Burkitt, 1962) have become a recognized method for elucidating the factors involved in neoplasia. Such surveys are particularly valuable in communities which are relatively static, since this reduces the number of variables to be considered. The present survey is a retrospective study of case records over the 14-year period 1954–67 at Ilesha where the author has worked for the last 3 years of the survey.

Ilesha is one of the larger Yoruba towns in the Southern part of the Western State of Nigeria. It is 1250 feet above sea level with a wet season from April to early November. The remainder of the year is dry, with scattered showers preceding or succeeding the rains. It is 75 miles East of Ibadan where a large Cancer Rate Survey was carried out between 1960 and 1963 (Edington and Maclean, 1965). There has been a mission hospital in Ilesha for over 50 years, but only permanent admission record charts since 1954.

During the period of the survey this was the only hospital serving Ilesha and surrounding areas. A total population of between half and three-quarters of a

TABLE I.—*Analysis of Tumours Seen in Ilesha* 1954–67

| No.* | 0–9 M | 0–9 F | 10–19 M | 10–19 F | 20–29 M | 20–29 F | 30–39 M | 30–39 F | 40–49 M | 40–49 F | 50–59 M | 50–59 F | 60–69 M | 60–69 F | 70+ M | 70+ F | Not Known M | Not Known F | All ages M | All ages F | Total | Hist-ology | Ilesha Town |
|---|---|---|---|---|---|---|---|---|---|---|---|---|---|---|---|---|---|---|---|---|---|---|---|
| 142 Salivary gland | — | — | — | — | 1 | — | — | — | — | 1 | 3 | — | — | — | — | — | — | — | 4 | 1 | 5 | 2 | — |
| 146–149 Pharynx | — | — | — | — | 2 | — | 1 | — | — | — | 1 | — | — | — | — | — | — | — | 4 | — | 4 | 1 | 1 |
| 151 Stomach | — | — | — | — | 1 | 2 | 3 | 3 | 10 | 5 | 11 | 7 | 7 | 13 | 5 | — | 2 | — | 39 | 30 | 69 | 20 | 38 |
| 152 Small intestine | — | — | 2 | — | — | — | — | — | 1 | — | — | — | 2 | — | — | — | — | — | 5 | — | 5 | 1 | 3 |
| 153 Colon | 1 | — | — | — | — | — | 1 | 1 | 2 | 2 | 4 | 2 | 1 | 1 | — | — | — | — | 9 | 6 | 15 | 3 | 7 |
| 154 Rectum | — | — | — | — | 2 | — | — | 1 | — | 3 | 3 | 2 | — | — | 1 | — | — | — | 6 | 6 | 12 | 8 | 7 |
| 155 Liver | — | — | — | 1 | 2 | — | 5 | 4 | 4 | 7 | 8 | 8 | 4 | 5 | 1 | — | — | — | 24 | 25 | 49 | 7 | 22 |
| 156 Gall baldder | — | — | — | — | — | — | — | — | — | — | — | 1 | — | — | — | — | — | — | — | 1 | 1 | — | — |
| 157 Pancreas | 1 | — | 1 | — | 1 | — | — | 1 | — | 1 | 1 | 1 | 2 | — | — | — | — | — | 6 | 3 | 9 | 4 | 4 |
| 160 Nose and nasal sinuses | — | — | — | — | — | 1 | — | — | 1 | 1 | 1 | — | — | 1 | 2 | — | — | — | 4 | 3 | 7 | — | 4 |
| 161 Larynx | — | — | — | — | — | — | — | — | — | — | 2 | — | — | — | — | — | — | — | 2 | — | 2 | 2 | — |
| 162–3 Lung and pleura | — | — | — | — | — | — | — | — | 1 | — | — | 1 | — | 2 | — | — | — | — | 1 | 3 | 4 | — | 3 |
| 170 Bone | 1 | — | — | — | 1 | — | — | 1 | 1 | — | 1 | — | — | 1 | — | — | — | — | 4 | 2 | 6 | 2 | 3 |
| 171 Conn. tissue | — | — | — | — | — | — | 2 | — | — | — | — | — | — | — | — | — | 1 | — | 3 | — | 3 | 1 | 1 |
| 172 Melanoma | — | — | — | 1 | — | 1 | 1 | — | 2 | — | 2 | — | 1 | — | — | 1 | — | — | 5 | 4 | 9 | 1 | 4 |
| 173 Skin | — | — | 1 | — | 1 | — | — | — | 2 | — | — | — | 1 | 1 | — | — | 1 | — | 6 | 1 | 7 | 3 | 2 |
| 174 Breast | — | — | — | — | — | 3 | — | 1 | — | 9 | — | 4 | — | 3 | — | — | — | — | — | 20 | 20 | 5 | 5 |
| 179 Penis and scrotum | — | — | — | — | — | — | — | — | — | — | — | — | — | — | — | — | — | — | — | — | — | — | — |
| 180 Cervix | — | — | — | — | — | — | — | 5 | — | 10 | — | 8 | — | 3 | — | — | — | 1 | — | 27 | 27 | 8 | 19 |
| 181 Chorionepithelioma | — | — | — | — | — | 6 | — | 4 | — | — | — | — | — | — | — | — | — | — | — | 10 | 10 | 6 | 4 |
| 182 Uterus | — | — | — | — | — | — | — | 1 | — | 1 | — | — | — | — | — | — | — | — | — | 2 | 2 | 1 | 1 |
| 183 Ovary | — | — | — | — | — | 3 | — | 1 | — | 4 | — | 2 | — | 1 | — | 2 | — | — | — | 13 | 13 | 5 | 8 |
| 185 Prostrate | — | — | — | — | — | — | — | — | 2 | — | — | — | 2 | — | — | — | — | — | 4 | — | 4 | — | 4 |
| 186 Testis | 1 | — | — | — | 2 | — | — | — | — | — | 1 | — | — | — | — | — | 1 | — | 5 | — | 5 | 3 | 2 |
| 188 Bladder | — | — | — | — | — | 1 | — | 1 | 1 | — | 1 | — | 2 | — | — | — | — | — | 3 | 3 | 6 | 1 | 3 |
| 189 Kidney | 3 | 2 | — | — | — | — | — | 1 | — | — | — | — | — | — | — | — | — | — | 4 | 2 | 6 | 3 | 2 |
| 190 Eye | 1 | 2 | — | — | — | — | — | — | — | — | — | — | 1 | — | — | — | — | — | 2 | 2 | 4 | 2 | 1 |
| 192 Brain | — | — | 2 | — | — | — | — | 1 | 1 | — | — | — | — | — | — | — | — | — | 3 | 1 | 4 | — | 2 |
| 193 Thyroid | — | — | — | — | — | — | — | — | — | — | — | — | 2 | — | — | — | — | — | 2 | — | 2 | 1 | 2 |
| 195 Abdominal cancer | — | — | 1 | — | 2 | — | 3 | 2 | 2 | 3 | 2 | 10 | 8 | 2 | 1 | — | — | 3 | 19 | 20 | 39 | 2 | 23 |
| 198 Secondary glands | — | — | — | — | — | — | — | — | 1 | — | 1 | — | — | — | — | — | — | — | 2 | — | 2 | 2 | 1 |
| 200 Lympho- and retic. sarcoma | 3 | — | 8 | 1 | 1 | 5 | 6 | 6 | 2 | 2 | 4 | 1 | 1 | 1 | 2 | 1 | — | — | 27 | 17 | 44 | 24 | 17 |
| 200[2] Burkitt tumour | 22 | 9 | 1 | 8 | — | — | — | — | — | — | — | — | — | — | — | — | 1 | — | 24 | 17 | 41 | 12 | 24 |
| 201 Hodgkins | — | — | — | — | 4 | — | 1 | 1 | 1 | — | — | — | — | — | — | — | — | 1 | 6 | 2 | 8 | 3 | 5 |
| 203 Multiple myedoma | — | — | — | — | — | — | — | — | — | — | — | — | 2 | — | — | — | — | — | 2 | — | 2 | 1 | 1 |
| 204 Lymphocytic leukaemia | 3 | — | — | — | — | — | 2 | — | — | 1 | 1 | — | — | — | — | — | — | — | 6 | 1 | 7 | 7 | 3 |
| 205 Myeloid leukaemia | 1 | 1 | — | — | — | — | 1 | 1 | 1 | 1 | — | 1 | 1 | — | — | — | 1 | — | 5 | 4 | 9 | 9 | 5 |
| 207 Leukaemia unspecified | — | — | 1 | — | — | — | — | — | 1 | — | 1 | — | — | — | — | — | — | — | 3 | — | 3 | 2 | 3 |
| | 37 | 14 | 17 | 11 | 20 | 22 | 26 | 36 | 36 | 51 | 48 | 48 | 37 | 34 | 12 | 4 | 7 | 5 | 239 | 226 | 465 | 152 | 234 |

* Classification numbers—International classification of Disease, WHO, 1967 Edition.

million people (95 per cent Yoruba tribe) were dependent upon its services. For the purpose of estimating incidence figures all patients from Ilesha township (with a population of 165,822 in the 1963 Nigerian Census) were separately analysed. There has been a specialist surgeon almost continuously on the staff during the 14 years. There were up to 40 beds available for surgical patients out of a total of 126.

### CASE MATERIAL

Hospital in-patient records form the basis of the present report with, in recent years, the pathology reports obtained for some out-patients who were not admitted. It is known that a number of terminal malignancies, particularly in the early years, were not admitted, but it has not been possible to trace their records. A few local patients treated in University College Hospital, Ibadan, and transferred back for follow-up were included.

The case notes of every patient admitted to the hospital during 14 years (72,862) were personally checked. Some originally diagnosed as malignant disease on doubtful grounds were rejected after reference to their subsequent history as revealed in the out-patients' records. Other patients not originally diagnosed were included if the records strongly suggested malignancy, confirmed by reference to follow-up records. The diagnoses in this group were (a) hepatoma, often previously recorded as " ascites " in the presence of a large irregular knobbly liver; (b) Burkitt's lymphoma, originally classified as atypical Cancrum oris or osteomyelitis of the jaw, affecting usually more than one quadrant and associated with abdominal masses or, in one case, paraplegia.

Patients admitted on more than one occasion are recorded as being admitted only once—during the first visit. No patient had two different malignant lesions. Most tumours not proved histologically were confirmed by operation. During the greater part of the survey period biopsies were only taken when clinical diagnosis was difficult. Among the diagnoses most liable to error are (a) lung and pleura, diagnosed on blood stained effusions with or without malignant cells; (b) abdominal cancer, a few cases of which may have been tuberculous, though to balance this several were discarded which may have been neoplastic; (c) Hodgkin's disease in the earlier years was an entirely clinical diagnosis, with lymphadenopathy in patients with a normal blood picture, some of whom were given anti-tuberculous treatment without response.

### *Selected Site—Details*

In Table I is summarized the complete analysis of all tumours.

Details of the commoner tumours follow.

#### *Stomach*

Full case notes were available for 62 of the 69 cases, the remaining seven were all confirmed histologically. In only 12 cases, all terminal or refusing surgery, was clinical evidence accepted without the supporting evidence of radiology, laparotomy or histology. Fifty of the 62 were subjected to laparotomy. With a single exception all the patients with gastric cancer were Yorubas. In 45 cases where the site was recorded, 28 involved the pyloric antrum (12 presented with pyloric stensois) 11 were in the body and 6 in the cardia, with extension into the oesophagus in 4. It is assumed that these 4 were initially gastric in origin.

(Compare ratios with Elebute *et al.* (1963) and Badoe (1966).) At operation resection was only possible in 16 out of 45. Table II shows the incidence by year of diagnosis.

TABLE II.—*Yearly Incidence of Carcinoma of Stomach*

| Year | 1954 | '55 | '56 | '57 | '58 | '59 | '60 | '61 | '62 | '63 | '64 | '65 | '66 | '67 |
|---|---|---|---|---|---|---|---|---|---|---|---|---|---|---|
| Number | 3 | 2 | 0 | 2 | 3 | 6 | 5 | 7 | 7 | 6 | 1 | 11 | 9 | 7 |

*Liver*

Only 16 of the total were proved at laparotomy; seven of these had biopsy. All others were clinical diagnoses with large knobbly livers; twenty had ascites and 9 had jaundice. Four were non-Yoruba, each from a separate tribe.

*Other gastrointestinal tract*

*Pharynx and Oesophagus.*—There was only one clinical diagnosis of carcinoma of the hypopharynx. No primary tumours of oesophagus were diagnosed (see note on cardia of stomach).

*Small intestine.*—All 5 were diagnosed at operation but only one biopsied (lymphoma). The others were similar macroscopically. All tumours involved the terminal ileum.

*Large intestine.*—Of the 15 only 2 were in the sigmoid colon and 1 in the descending colon. There were 6 in the caecum (1 proved carcinoma and 2 reticulosarcoma), 2 in the hepatic flexure, 1 in the splenic flexure and 3 in the transverse colon. A similar high incidence of right-sided colon lesions has received comment from Davies *et al.* (1964) in Uganda with 46 per cent (compared with 50 per cent here) and Williams and Edington (1967) in Ibadan with 55 per cent.

*Rectum.*—Eight of the 12 were biopsied. Only 1 agreed to surgery.

*Pancreas.*—Nine were found at operation but only 4 were biopsied. The others were large tumours suggesting lymphosarcoma.

*Lymphoreticular system (except Burkitt)*

All the leukaemias were diagnosed on the blood picture. Multiple myeloma was diagnosed on bone marrow aspiration and/or typical radiological and biochemical findings. Hodgkin's disease was a clinical diagnosis in earlier years, though the last 3 were confirmed histologically. Lymphosarcoma was confirmed by laparotomy in all abdominal cases, though only recently have all been biopsied.

*Burkitt's lymphoma*

There was a total of 41, with male to female ratio 24 : 17. In the first 7 years there were 13 compared with 28 in the second 7 years (15 in the past 3 years). Nineteen had typical jaw tumours. Nine girls had ovarian tumours. The 12 tumours biopsied were in cervical glands, testis, thyroid and retroperitoneum. Five clinically diagnosed cases with raised intracranial pressure and/or paraplegia are included.

Table III shows the distribution with age, in patients coming from Ilesha township. The standard population for Africa, as suggested by Knowelden and Oettlé (1962) is used to give the estimated population at risk in each age group per annum.

TABLE III.—*Burkitt's Lymphoma in Ilesha Township*

| Age (Years) | 0–4 | 5–9 | 10–14 |
|---|---|---|---|
| Numbers | 5 | 14 | 4 |
| Estimated population | 15,000 | 15,000 | 15,000 |
| Rate/100,000 | 2·4 | 6·7 | 1·9 |

The age specific rate (both sexes/100,000) up to 5 years accords with the picture given by Edington and Maclean (1964) in Ibadan, who found a peak in the 5–9 years age group (15 compared with 6·7/100,000). In the 0–14 age group there were 66 tumours in the whole series, Burkitt's lymphoma representing 62 per cent (70 per cent in Ibadan). These figures emphasize the importance of this tumour.

*Female genital tract*

Only 2 tumours of the uterine body were seen—one confirmed histologically. There were 27 carcinomas of the cervix, almost all of which were inoperable. The advanced stage of disease in those presenting suggests that other sufferers do not come to hospital. The group of chorionepitheliomas is more truly representative. It is considered locally to be worth a financial sacrifice, if necessary, in order to continue childbearing. Tumours of the ovary (usually adenocarcinoma) were confirmed by operation. Burkitt's tumour of the ovary is included under Burkitt's lymphoma.

*Breast*

Most of the 20 cases were far advanced when seen. One patient with an ulcerated lesion presented with tetanus. Another patient developed tetanus 2 days after removal of the tumour.

*Miscellaneous sites*

*Skin tumours* were not common. Epitheliomas were diagnosed in the early years (as were all but one of the melanomas.) Only 4 were biopsied. Melanomas were typically on the heel. Albinos seem to be particularly prone to recurrent facial epitheliomas. No Kaposi's sarcomas were diagnosed.

*Urinary tract.*—All renal tumours except one were in children. Those examined histologically were Wilms' tumour but others in retrospect may have been Burkitt's lymphoma. Bladder tumours were all operative diagnoses—a small number in view of the relatively high incidence of urinary schistosomiasis in clinical practice.

*Male genital tract.*—Prostatic carcinoma was a purely clinical diagnosis with neither biopsy nor biochemical confirmation, though several had a favourable response to oestrogens. There were 6 testicular tumours. One was a Burkitt's lymphoma and is classified under that heading. Two were seminomas, 2 teratoma and one choriocarcinoma. There were no penile carcinomas (all males have circumcision).

*Thyroid.*—There were only 2 anaplastic tumours and 1 Burkitt's lymphoma. As multinodular goitres are common this low number is surprising. Several tumours which appeared to be malignant clinically were found at biopsy to be thyroiditis.

*Nasal sinuses.*—When childhood lymphomas were excluded only 7 remained. These were clinically maxillary carcinoma or sarcoma.

### *Statistical Data*

### *Numbers seen*

The recent increase in numbers, most of which were histologically confirmed, reflects an increased total number of admissions. The percentage of admissions due to cancer was steady throughout the years (Table IV).

TABLE IV.—*Yearly Numbers of Malignant Disease with Percentage of Total Hospital Admissions*

| Year | 1954 | '55 | '56 | '57 | '58 | '59 | '60 | '61 | '62 | '63 | '64 | '65 | '66 | '67 |
|---|---|---|---|---|---|---|---|---|---|---|---|---|---|---|
| Number | 18 | 18 | 18 | 17 | 22 | 34 | 27 | 29 | 36 | 53 | 34 | 60 | 57 | 49 |
| Percentage | 0·8 | 0·9 | 0·6 | 0·5 | 0·5 | 0·8 | 0·5 | 0·5 | 0·7 | 0·8 | 0·5 | 0·7 | 0·8 | 0·7 |

In Table I the total for each site is recorded. Under Burkitt's lymphoma are included tumours of all sites. Many of the " abdominal cancer " cases were probably stomach or liver in origin. Lympho- or reticulosarcoma mainly or entirely involving a specific organ is included under that site, *e.g.* small intestine, caecum and pancreas, as biopsy to distinguish from carcinoma was not always done. Had this histological type from all sites been included under one heading it would have been exceeded only by carcinoma of stomach and hepatoma.

### *Relative ratios*

Figures for each site are shown in Table V. Those referring to cervix uteri and breast, because of their selection, were an underestimate of the ratio in the community; consequently other relative ratios have been inflated especially in females.

TABLE V.—*Relative Ratio Frequencies for Most Common Malignancies*

| | Male (239) | | Female (226) | | Total (465) | |
|---|---|---|---|---|---|---|
| | No. | RRF% | No. | RRF% | No. | RRF% |
| 1. Stomach | 39 | 16·3 | 30 | 13·2 | 69 | 14·8 |
| 2. Liver | 24 | 10·0 | 25 | 11·0 | 49 | 10·5 |
| 3. Retic. & lympho-sarcoma | 27 | 11·3 | 17 | 7·4 | 44 | 9·4 |
| 4. Burkitt | 24 | 10·0 | 17 | 7·4 | 41 | 8·6 |
| 5. Abdominal cancer (uncertain origin) | 19 | 7·9 | 20 | 8·8 | 39 | 8·1 |
| 6. Cervix uteri | — | — | 27 | 11·9 | 27 | 5·6 |
| 7. Breast | — | — | 20 | 8·8 | 20 | 4·3 |
| 8. Leukaemias | 14 | 5·8 | 5 | 2·2 | 19 | 4·1 |
| 9. Colon | 9 | 3·8 | 6 | 2·6 | 15 | 3·2 |
| 10. Ovary | — | — | 13 | 5·7 | 13 | 2·8 |
| 11. Rectum | 6 | 2·5 | 6 | 2·6 | 12 | 2·6 |
| 12. Chorio-carcinoma | — | — | 10 | 4·4 | 10 | 2·1 |

As comparison of tumour frequencies between different series highlights local characteristics, Table VI compares English-speaking West African figures.

Carcinoma of the stomach is the one tumour with a greater incidence in Ilesha than elsewhere in West Africa. Camain's (1954) experience in French-speaking West Africa was 43 in 1884 cases—a frequency of 2·3 per cent. Similarly 2·8 per cent in Kampala Cancer Registry and 4·3 per cent in Mengo hospital reflect a ratio corresponding to general West African experience (Davies *et al.*, 1964). The only comparable high relative ratio figures in Africa have been reported from

TABLE VI.—*Comparison of Relative Ratio Frequencies from English-speaking West Africa*

| | Elmes and Baldwin (1947) Lagos (Biopsy) (1000) | Duncan (1968) Lagos (Clinical) (286) | Edington and Maclean (1965) Ibadan (Biopsy and clinical) (1920) | Berry (1964) N. Nigeria (Biopsy) (296) | Edington (1956) Ghana (Biopsy) (1193) | Mulligan Present series (465) |
|---|---|---|---|---|---|---|
| Stomach | 2·2 | 4·5 | 4·4 | 5·4* | 3·6 | 14·8 |
| Liver | 8·1 | 9·4 | 7·2 | 2·7 | 7·6 | 10·5 |
| Retic. and lympho-sarcoma | 19·0† | 7·7 | 8·8 | — | 5·7† | 9·4 |
| Burkitt | — | 3·4 | 8·9 | 5·7 | — | 8·6 |
| Cervix | 6·8 | 3·5 | 9·4 | 10·4 | 4·6 | 5·6 |
| Breast | 8·4 | 8·7 | 6·9 | 5·4 | 5·4 | 4·3 |
| Colon and rectum | 1·4 | 4·2 | 2·7 | — | 1·7 | 5·8 |

* All gastro-intestinal tumours.
† Includes leukaemia and Burkitt tumour.

Eastern Kivu in Congo with 11·5 per cent (Clemmesen *et al.*, 1962), at Ndolage in the extreme North-west of Tanzania with 16 per cent (quoted by Buckley, 1967) and from Western Kenya 16·5 per cent (Kisia and Burkitt, 1968).

*Crude rates*

In spite of conventional Christian tradition to the contrary, some of the churches in Ilesha, which is predominantly Christian, have tended to emphasize " faith healing " and refused to use the available medical facilities.

This tendency is more pronounced among the older age group, and is likely to have had a selective influence on tumour statistics because of the age group involved. It is estimated—based on the number of church assemblies adhering to the belief—that approximately 30 per cent of the population at risk were excluded from hospitalization. This allows an approximate correction factor for the figures obtained in Table VII.

TABLE VII.—*Crude Incidence of Malignant Disease Per* 100,000 *Per Annum* (*Ilesha Township*)

| | Total | Incidence | Corrected incidence |
|---|---|---|---|
| Carcinoma stomach | 38 | 1·81 | 2·6 |
| Hepatoma | 22 | 1·05 | 1·5 |
| Carcinoma cervix | 19 | 0·90 | 1·3 |
| Retic and lymphosarcoma | 17 | 0·81 | 1·2 |
| Burkitt's lymphoma | 24 | 1·14 | 1·6 |
| Abdominal cancer | 23 | 1·10 | 1·6 |
| All tumours | 234 | 11·14 | 18·8 |

In estimating these figures the 1963 Nigerian census, reporting 165,822 in Ilesha, is used for calculation. While the population for 1954 to 1963 was less than this, it is known that the population since the census has grown steadily. For ease of calculation a figure of 150,000 was used as the average population per annum over the 14 years. This is possibly an overestimate producing lower incidence figures. The figures even when corrected for religious bias, are low compared with Ibadan which has a crude annual incidence of 45 per 100,000 (though the Ibadan figures are based on the provisional figures for the 1962 census and not the more accurate 1963 census which gave a larger population). As yet unpublished data from Imesi research village, 25 miles from Ilesha, where accurate statistics are available, show that the annual death rate for known or suspected cancer was 129 per 100,000 during the same period. Though not strictly comparable this does suggest that the present figures are a gross underestimate of the true situation.

*Age specific rates*

These have only been calculated for Burkitt's lymphoma (Table III) and carcinoma of stomach (Table VIII)—the latter because the numbers were greater than for other tumours and because relative ratio frequencies were high compared with other African reports.

TABLE VIII.—*Age Specific Rates for Stomach Carcinoma*/100,000 *Population at Risk*

| Age (in years) | 35–39 | 40–44 | 45–49 | 50–54 | 55–59 | 60–64 |
|---|---|---|---|---|---|---|
| Male | 1·1 | 3·1 | 7·3 | 10·5 | 12·2 | 17·5 |
| Female | 1·1 | 3·1 | 2·1 | 6·2 | 9·6 | 20·9 |

In estimating the population at risk in each age group an Arbitrary Standard Population for African Races is used (Knowelden and Oettlé, 1962). This is similar to the actual figures in Ibadan (Edington and Maclean, 1965). Following the suggestion of Doll (1968) " only those age groups are analysed which are useful for comparison with reports from other countries and cultures ". The purpose of this comparison is to determine current differences in the presence of possible carcinogenic factors.

These results are similar to figures from Ibadan (Edington and Easmon, 1965) and, within the three decades analysed, closely parallel the incidence in Connecticut, U.S.A. (Doll, 1968).

### DISCUSSION

In East Africa there have been several studies of cancer incidence published from district mission hospitals (Williams, 1966; Eshleman, 1966; Buckley, 1967; Kisia and Burkitt, 1968). These have shown local variations. Up to the present no such reports have originated from similar hospitals in West Africa. This is the first, it is hoped, of a number on the west coast. Though not having the large numbers of a city or central pathology service, it probably reflects more clearly the actual situation in the country. It is dangerous and false to extrapolate experience in a large city to the whole country or even to the not so distant hinterland. Ibadan and Ilesha figures can be compared in Table VI. Oettlé (1966) has shown that there are variations between town and country. Now that certain differences between Ilesha and Ibadan are apparent a tumour registry is being kept and a prospective study commenced.

In Ijesha Division of which Ilesha is the administrative centre, 95 per cent of the people are Yorubas (final analysis 1952 Nigerian census). Hospital admissions reflect this ratio, with only 4·5 per cent of all patients being non-Yoruba. No tribal differences have been noted among the tumour admissions.

Admission policy is relevant to the present study based mainly on hospital admissions. In the earlier years advanced tumours were not always admitted. They were referred directly to the teaching hospital in Ibadan, though most case summaries have been lost. Until 1957 everyone paid for treatment; thereafter all children up to 18 years of age were treated free of charge, food excepted. Fifty-five per cent of all admissions were under 18 years of age. A further 15 to 20 per cent of the total were admitted to the maternity department. These figures qualify the rather low malignancy rate per 100 admissions. This varied between 0·5 and 0·9 per cent (see Table IV). This is less than the 0·91 from Mengo (Davies *et al.*, 1964) and the average 1 per cent mentioned by Buckley (1967).

During the period under survey this area was among the most prosperous in Nigeria. Financial considerations were thus less weighty, certainly for males, than in many developing countries.

Comparison with Ibadan township shows interesting contrasts. In Ibadan, with potentially free treatment and the greater depersonalization of city life affecting attitudes to mastectomy, and with radiotherapy facilities attracting cervical cancer patients, carcinoma of cervix and breast are the commonest tumours seen. In Ilesha both social pressures and the financial difficulties for females have militated against a true picture of these conditions. Burkitt's lymphoma shows a similar pattern to Ibadan both in relative ratio and in age specific rates (Table II) though the increase with age is not so marked in Ilesha. Lymphosarcoma is similar. Most significant is the marked difference in carcinoma of stomach where frequency ratios are double in the male and treble in the female when compared with Ibadan (16·3 : 8·5 for males and 13·2 : 4·2 for females). Age specific analysis does not confirm this difference and suggests some degree of selection. The incidence of duodenal ulceration, but not gastric ulceration, is high in this area. Its treatment comprises a major part of abdominal surgery. Direct questioning does not indicate any unusual intake of dried smoked fish compared with other parts of Yoruba land. Kola nuts are commonly chewed by people of both sexes and all ages. Cigarette smoking has only recently gained acceptance. Palm wine is consumed in large quantities but illicit gin and home-brewed beers are rare.

Because duodenal ulceration was known to be common, there was an undoubted interest by doctors in symptoms referable to the upper gastro-intestinal tract. Several cases of pyloric stenosis have had operation, on the assumption that chronic duodenal ulcer fibrosis was the aetiology, only to discover an early pyloric canal tumour. This factor in itself cannot account for an incidence two to three times that in Ibadan, where duodenal ulcer and its complications are also common. No causative factors are known but the absence of oesophageal tumours, the low incidence of benign gastric ulcers and the high duodenal ulcer rate are all significant.

It is to be noted that no case of lip, tongue or oesophageal carcinoma or Kaposi's sarcoma was found. This is in contract to several East and South African reports, although consistent with the West African series. No case of carcinoma of the penis was seen, which is not surprising since most males are radically circumcised soon after birth. Carcinoma of the cervix however was common. In East Africa epithelioma developing in the scar of old tropical ulcers is very common, but no case is reported in this series.

Thanks are due to Professor G. M. Edington and Dr. A. O. Williams of the Department of Pathology, University of Ibadan, both of whom made valuable suggestions while the data were being analysed. Willing help from the department over the years has been much appreciated. The enthusiasm and encouragement of Mr. D. P. Burkitt of the Medical Research Council External Scientific Staff, London, has been an inspiration to complete the present study and initiate a tumour registry for further information.

### REFERENCES

BADOE, E. A.—(1966). *W. Afr. med. J.*, **15**, 181.
BERRY, C. G.—(1964) *Br. med. J.*, ii, 668.
BUCKLEY, R. M.—(1967) *E. Afr. med. J.*, **44**, 465.
BURKITT, D. P.—(1962) *Br. med. J.*, ii, 1019.
CAMAIN, R.—(1954) *Bull. Soc. Path. exot.*, **47**, 614.
CLEMMESEN, J., MAISIN, J. AND GIGASE, P.—(1962) Preliminary Report on Cancer in Kivu and Ruanda-Urundi. University of Louvain, Institute of Cancer.
DAVIES, J. N. P., ELWES, S., HUTT, M. S. R., MTIMAVALYE, L. A. R., OWOR, R. AND SHAPER, L.—(1964) *Br. med. J.*, i, 259 and 336.
DOLL, R.—(1968) 'Cancer in Africa.' Nairobi (East African Publishing House) pp. 105–110.
DUNCAN, J. T. K.—(1968) *W. Afr. med. J.*, **17**, 96.
EDINGTON, G. M.—(1956) *Br. J. Cancer*, **10**, 595.
EDINGTON, G. M. AND EASMON, C. O.—(1965) 'Cancer of the Alimentary Tract in Africa.' UICC Symposium, Geneva, 1965.
EDINGTON, G. M. AND MACLEAN, C. M. H.—(1964) *Br. med. J.*, i, 264.—(1965) *Br. J. Cancer*, **19**, 471.
ELEBUTE, E. A., NGU, V. A., MAINWARING, A. R. AND ERUCHALU, R. C.—(1963) *W. Afr. med. J.*, **12**, 11.
ELMES, B. G. T. AND BALDWIN, R. B. T.—(1947) *Ann. trop. Med. Parasit.*, **41**, 321.
ESHLEMAN, J. L.—(1966) *E. Afr. med. J.*, **43**, 274.
KISIA, I. A. AND BURKITT, D. P.—(1968) *E. Afr. med. J.*, **45**, 706.
KNOWELDEN, J. AND OETTLÉ, A. G.—(1962) Unpublished data used by Davies, J. N. P., Wilson, B. A. and Knowelden, J. (1966) *Lancet*, ii, 328.
OETTLÉ, A. G.—(1966) *S. Afr. J. med. Sci.*, **31**, 21.
SMITH, J. AND ELMES, B. G. T.—(1934) *Ann. trop. Med. Parasit.*, **41**, 321.
WILLIAMS, A. O. AND EDINGTON, G. M.—(1967) *Dis. Colon Rectum*, **10**, 301.,
WILLIAMS, E. H.—(1966) *E. Afr. med. J.*, **43**, 200.